# Serological assessment of pediatric parasite exposure in two Senegalese districts using multiplex serology

**DOI:** 10.1016/j.crpvbd.2025.100320

**Published:** 2025-09-19

**Authors:** Helena Brazal Monzó, Santiago Rayment Gomez, Doudou Sow, Aminata Colle Lo, Marie Pierre Diouf, Amadou Seck, Ibrahima Mbaye, Elhadji Babacar Fall, Catriona Patterson, Seyi Soremekun, Isaac A. Manga, Cheikh Cissé, Awa Diouf, Ndéye Aida Gaye, Kevin K.A. Tetteh, Alex Loukas, Brian Greenwood, Jean Louis A. Ndiaye, Chris Drakeley, Muhammed O. Afolabi

**Affiliations:** a Department of Infection Biology, London School of Hygiene and Tropical Medicine, Keppel Street, London, WC1E 7HT, UK; b Department of Population Health, London School of Hygiene and Tropical Medicine, Keppel Street, London, WC1E 7HT, UK; c Service de Parasitologie-Mycologie, Université Gaston Berger de Saint-Louis, BP 234, Saint-Louis, Senegal; d Service de Parasitologie-Mycologie, Université Cheikh Anta Diop, Dakar-Fann, BP 5005, Dakar, Senegal; e Service de Parasitologie-Mycologie, Université de Thies, BP 967, Thiès, Senegal; f FIND Global Health Campus, Chemin du Pommier 40, Le Grand-Saconnex, 1218, Geneva, Switzerland; g Australian Institute of Tropical Health and Medicine, James Cook University, McGregor Road, Cairns, QLD, 4870, Australia; h Department of Disease Control, London School of Hygiene and Tropical Medicine, Keppel Street, London, WC1E 7HT, UK

**Keywords:** Malaria, Helminths, Enteric protozoa, Seroprevalence

## Abstract

Although pediatric parasitic diseases cause significant morbidity and mortality in regions with high rates of co-infection, this overlap may offer opportunities for integrated control strategies. This study aimed at a serological assessment of exposure to multiple parasitic infections among children aged 1–14 years in two Senegalese districts, Saraya (Kédougou Region) and Diourbel (Diourbel Region), to inform integrated control strategies. We analysed 883 dried blood spot samples. A multiplex bead-based immunoassay quantified IgG antibody against *Plasmodium falciparum*, helminths (*Necator americanus*, *Schistosoma mansoni*, *Strongyloides stercoralis*, *Taenia solium*), and intestinal protozoa (*Cryptosporidium parvum*, *Giardia duodenalis*) as proxies for single- and multiple-pathogen exposure. Multivariable logistic regression identified risk factors for seropositivity. Recent malaria exposure was identified in 11% of children, while 42% showed evidence of historical exposure. Helminth seroprevalence ranged between 0.1% and 7.2%, whereas *Cryptosporidium parvum* and *Giardia duodenalis* seroprevalence values were 19.0% and 7.4%, respectively. Co-exposures to malaria and other parasites ranged from 9.4% to 18.0%. School-aged children exhibited higher seroprevalence rates for historical exposure to *P. falciparum* and *S. stercoralis* compared to pre-school children, while *G. duodenalis* was more seroprevalent in pre-school children. Saraya exhibited higher seroprevalence for historical *P. falciparum* and *G. duodenalis* exposure. Rare/never handwashing before meals, shorter travel time to a water source (< 10 min, likely reflecting residence near shared or surface water rather than improved household taps), and frequent contact with any waterbodies (daily/weekly) were associated with higher odds of parasite seropositivity. While seasonal malaria chemoprevention appears suitable, the low helminth seroprevalence coupled with substantial protozoan exposure suggests that current integrated interventions may require re-evaluation and enhancement.

## Highlights

• Serological assessment of exposure to multiple parasitic infections among children in two districts in Senegal.
• IgG serology showed low helminth exposure, supporting elimination efforts.
• *Plasmodium falciparum* exposure remained moderate among participants, mostly in Saraya District.
• High enteric protozoa seropositivity suggests need to explore interventions.
• Poor handwashing, and close/frequent water contact raised seropositivity odds.

## Graphical abstract

**Figure:**
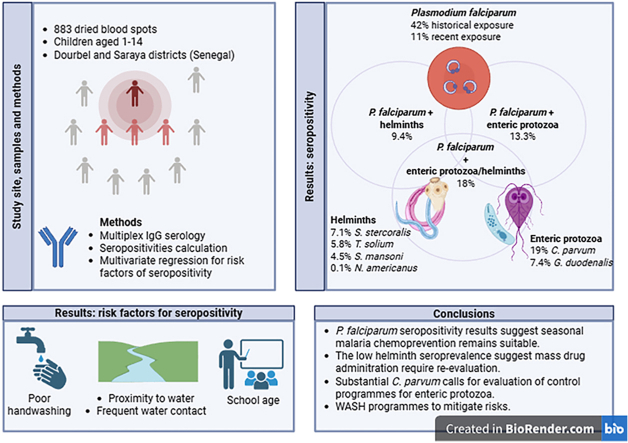

## 1 Introduction

Despite extensive efforts to control malaria, *Plasmodium falciparum* caused approximately 263,000 new cases worldwide in 2023, with sub-Saharan Africa (SSA) bearing over 90% of cases and deaths (WHO, 2023). This region also suffers a high burden of other parasitic infections, including intestinal protozoa (e.g. *Cryptosporidium* spp., *Giardia duodenalis* (syn. *Giardia lamblia*)) (Squire and Ryan, 2017), and diverse helminths such as soil-transmitted nematodes, food-borne cestodes and schistosomes (Degarege et al., 2016; Madinga et al., 2017; Afolabi et al., 2021, 2022).

The health impact of exposure to multiple parasites remains uncertain (Fernández-Niño et al., 2012). Some studies link helminth infections with increased malaria risks, higher parasite densities, severe anemia, and adverse pregnancy outcomes (Yatich et al., 2010; Fernández-Nino et al., 2012; Dassah et al., 2023), while others suggest protective effects, such as *A. lumbricoides* reducing malaria severity (Brutus et al., 2007). Other research projects found no significant effects of co-infections (Fernández-Niño et al., 2012). These discrepancies may be attributed to differences in genetics, immunity, and parasite burden, highlighting the complexity of multi-parasite exposure. Additionally, intestinal protozoa such as *C. parvum* and *G. duodenalis* cause severe diarrhoeal disease leading to dehydration and malnutrition, further exacerbating health burdens in populations already affected by *P. falciparum* (Coutinho et al., 2008; Reiner et al., 2022). Together, malaria and diarrhoeal diseases are leading contributors to disability-adjusted life years (DALYs) in children under five years of age, underscoring their major contribution to childhood morbidity and mortality (Reiner et al., 2022).

Although the precise interactions among these pathogens remain unclear, their combined negative impact is undeniable. In addition to mortality, infections with *Cryptosporidium*, *Giardia*, helminths, and *Plasmodium* are linked to impaired cognitive and physical development in children (Berkman et al., 2002; LaBeaud et al., 2015). Nevertheless, this geographical overlap might enable integrated interventions targeting multiple pathogens, thereby optimizing resource allocation. The co-endemicity of malaria, helminths, and intestinal protozoa in SSA suggests that integrated control strategies could be more effective and efficient, necessitating better characterization of exposure patterns. Our previous study in Senegal found low PCR-detected prevalence and co-infection rates for malaria, schistosomiasis, and soil-transmitted helminths, suggesting that regional elimination may be within reach (Afolabi et al., 2023b). In contrast, moderate levels of enteric protozoa highlighted gaps in current control efforts (Afolabi et al., 2023b). To build on these findings, the present study assessed past exposure to multiple parasites in the same individuals using bead-based detection of IgG antibodies against malaria (*P. falciparum*), helminths (*N. americanus*, *S. mansoni*, *S. stercoralis*, and *T. solium*), and intestinal protozoa (*C. parvum* and *G. duodenalis*). This study aims to: (i) estimate IgG antibody seroprevalence as a marker of exposure history to *P. falciparum*, soil- and food-transmitted helminths, schistosomes, and food- and water-borne protozoa in children aged 1–14 years; and (ii) identify demographic and environmental risk factors to inform targeted and integrated intervention programmes, including Seasonal Malaria Chemoprevention (SMC) combined with anthelmintics (Afolabi et al., 2023a, 2023c), and evaluation of tinidazole for intestinal protozoa management.

## 2 Materials and methods

### 2.1 Study sites and samples

**Fig. 1:**
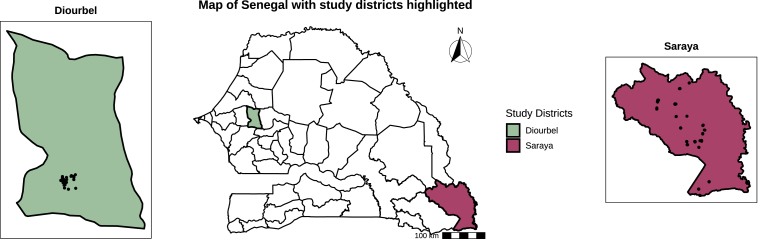
Map of Senegal including inset maps of both study districts, with points indicating coordinates of sample collection.

Samples were collected between June and November 2021 in two districts, Diourbel and Saraya. Diourbel is a suburban town 150 km east of Dakar and the capital of Diourbel Region. Saraya is a rural town in the Kédougou Region (Fig. 1). The climate in both areas is Sudano-Sahelian, with a rainy season (July-October) and a dry season (November-June).

We analysed 883 dried blood spot (DBS) samples collected on Whatman 3MM paper from children aged 1–14 years; samples were stored at 4 °C with desiccant until processing. The age distribution, behavioural and demographic variables are provided in Supplementary Table S1. Of the 883 DBS, 347 were from Diourbel and 536 from Saraya. The sampling protocol is described in Afolabi et al. (2023b).

In both districts, control interventions for soil-transmitted helminths (STH) and schistosomiasis have been implemented annually since 2014 among school- and pre-school-aged children (ESPEN, 2020; MSASS, 2022). Seasonal malaria chemoprevention (SMC) with sulfadoxine-pyrimethamine plus amodiaquine was introduced in 2013 in Saraya and in 2019 in Diourbel, achieving high coverage in both districts (Cissé et al., 2016; MSASS, 2022).

### 2.2 Antigen panel and bead coupling

Luminex MagPlex® COOH microspheres were conjugated to a panel of 20 recombinant antigens selected based on prior evaluations of immunogenicity, specificity, and sensitivity (Supplementary Table S2). Antigens were expressed at the London School of Hygiene & Tropical Medicine (LSHTM), the United States Centers for Disease Control and Prevention (US CDC), or James Cook University (JCU; Australia). LSHTM- and US CDC-derived antigens were expressed as described previously (Rogier et al., 2019; Wu et al., 2019), and JCU antigens were expressed as recombinant His-tagged proteins.

This antigen panel represented helminths, enteric protozoa, and *P. falciparum* malaria (Supplementary Table S2). In addition, glutathione-S-transferase (GST) was included as an internal control to assess cross-reactivity with GST-tagged proteins. Diphtheria toxoid (*Corynebacterium diphtheriae*) and tetanus toxoid (*Clostridium tetani*) were included as internal controls for total IgG antibody content, given high vaccination coverage in the study population.

US CDC-expressed antigens were conjugated at US CDC following earlier methods (Rogier et al., 2019), and the remaining antigens were conjugated at LSHTM using previously optimised antigen concentrations and established protocols (Wu et al., 2019). All antigens were assayed simultaneously in a single multiplex run per sample.

### 2.3 Sample elution and IgG quantification

Three-millimetre (3-mm) punches from each DBS were eluted overnight at 1:400 in Buffer B (1× PBS, 0.05% Tween-20, 0.5% BSA, 0.02% sodium azide, 0.1% casein, 0.5% polyvinyl alcohol (PVA), 0.5% polyvinylpyrrolidone (PVP)) supplemented with 15.25 μg/ml E*. coli* lysate to reduce non-specific background against antigens expressed in *E. coli*. For each DBS, 399 μl of Buffer B were added, using the same buffer batch for all samples.

Eluates were assayed simultaneously for IgG responses using the Luminex MAGPIX® platform, as described previously (Wu et al., 2019). Median fluorescence intensity (MFI) was recorded for each analyte and sample. Serial dilutions of a hyperimmune Tanzanian serum pool (CP3) were used to generate standard control curves. Anti-malaria (*P. falciparum*) human serum (NIBSC; 1st WHO Reference Reagent, 10/198) served as a positive control for *P. falciparum* IgG. Positive-control sera for helminths and enteric protozoa were provided by the US CDC. Sera from UK residents with no travel history, supplied by the UK Health Security Agency (UKHSA), were used as negative controls.

### 2.4 Quality control

Between-plate variance was assessed by plotting each dilution point of the standard curves on Levey-Jennings charts. Plates were repeated if more than two dilution points for more than two antigens deviated by > 2 standard deviations of the mean. GST signals were reviewed across plates to confirm MFIs < 1000, indicating minimal cross-reactivity. Diphtheria toxoid and tetanus toxoid MFIs were also reviewed per plate to verify sample quality, ensuring that most samples had signals > 5000 MFI and that signals were consistent across plates. To standardize background noise, the mean blank value across all plates was subtracted from each sample’s median MFI.

### 2.5 Calculation of cut-off values and antibody levels

Antigens were classified by pathogen type and, for *P. falciparum*, by exposure category (historical/cumulative, recent, and protective) (Supplementary Table S2).

Serostatus was defined using two approaches: (i) a cut-off set at three standard deviations above the mean IgG level from UKHSA immune-naive samples; and (ii) a finite mixture model (FMM) implemented with the R package *mixtools* (version 4.2.1), with the cut-off defined as three standard deviations above the mean of the lower Gaussian component (seronegative group). For each antigen, the cut-off was selected based on biological plausibility, consistency with prior work, and agreement with PCR data.

To summarize antibody responses, we computed the arithmetic mean of the log10-transformed MFIs for antigens with similar immunogenicity representing the same pathogen, or specific exposure markers (e.g. recent *vs* historical exposure), to increase the breadth of immune responses captured. Cutt-off selected and antibody level criteria can be found in Supplementary Table S2.

### 2.6 Multiple exposure criteria

Co-exposure was defined as an individual testing seropositive for *P. falciparum* and at least one other pathogen type. This included *P. falciparum* with any helminths (*N. americanus*, *S. mansoni*, *S. stercoralis*, and/or *T. solium*), *P. falciparum* with any intestinal protozoan (*C. parvum* and/or *G. duodenalis*), or *P. falciparum* with any water, sanitation, and hygiene (WASH)-associated pathogen. WASH-associated pathogens encompassed all helminths and intestinal protozoa.

### 2.7 Statistical analysis

We compared seroprevalence across sites, age groups, and sex using Pearson’s chi-square tests, applying Fisher’s exact tests when any expected cell count was < 5. Statistical significance was assessed at *P* < 0.05 and confirmed with non-overlapping 95% confidence intervals (CI). Age groups were categorized as preschool-aged children (5 years or younger) and school-age children (older than 5 years).

To infer potential shared risk factors, we assessed pairwise correlations between antibody levels using Pearson’s correlation coefficient. Associations between specific risk factors and (i) seropositivity to a single pathogen (*vs* seronegativity) and (ii) multiple-pathogen seropositivity (*vs* single-pathogen seropositivity or seronegativity) were examined using multivariable logistic regression with backward elimination, selecting the best-fitting model using Akaike’s Information Criterion (AIC). To control the family-wise error rate (FWER) while preserving statistical power, we adjusted *P*-values using the Holm-Bonferroni method. Explanatory variables were selected from epidemiological questionnaires developed by Afolabi et al. (2023b), based on prior knowledge of factors likely to influence exposure to the pathogens under study. Categorical variables were re-coded as dummy variables where appropriate.

Categories representing less than 10% of the sample were excluded unless their inclusion in another variable was epidemiologically relevant. For predictors with high pairwise association (Cramér's V > 0.5), only the most epidemiologically relevant variable was retained. A complete list of final explanatory variables is provided in Supplementary Table S1.

## 3 Results

### 3.1 Exposure to single pathogens

Across both sites, seroprevalence was 18.8% (95% CI: 16.2–21.4%) for *C. parvum,* 7.4% (95% CI: 5.6–9.1%) for *G. duodenalis,* 7.1% (95% CI: 5.4–8.8%) for *S. stercoralis,* 5.8% (95% CI: 4.2–7.3%) for *T. solium,* and 4.5% (95% CI: 3.2–5.9%) for *S. mansoni*. One individual was seropositive for *N. americanus*. For *P. falciparum*, 41.7% (95% CI: 38.4–44.9%) of the participants were seropositive for historical exposure, 12.2% (95% CI: 10.1–14.4%) for partial-protection markers, and 11.2% (95% CI: 9.1–13.3%) for recent exposure.

**Fig. 2:**
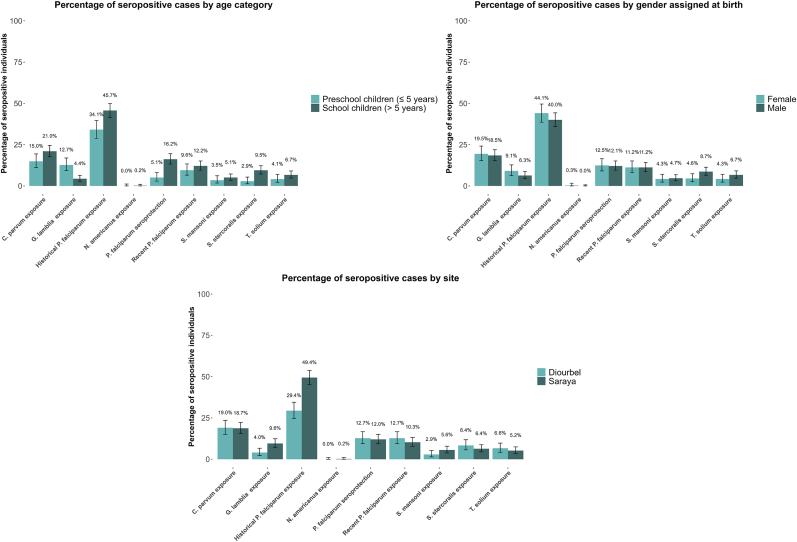
Seroprevalence observed amongst 883 participants at study sites by age, site, and gender. Bars indicate 95% confidence intervals.

School-aged children had higher seroprevalence for historical *P. falciparum*, partial *P. falciparum* protection, and *S. stercoralis* than pre-school children (Fig. 2, Supplementary Table S3). *Giardia duodenalis* seroprevalence was higher among preschool children (Fig. 2, Supplementary Table S3). Saraya showed higher seroprevalence for historical *P. falciparum* and *G. duodenalis* than Diourbel (Fig. 2, Table 1). No sex differences were observed (Fig. 2, Supplementary Table S4).

**Table 1:** Summary of participants who tested seropositive to single pathogen types and who were exposed to more than one pathogen type by study site. Groups of co-exposure were: exposed to *P. falciparum +* WASH-related pathogens (*N. americanus*, *S. mansoni*, *S. stercoralis*, *T. solium*, *C. parvum* and *G. duodenalis*); exposed to *P. falciparum +* helminths (*N. americanus*, *S. mansoni*, *S. stercoralis*, *T. solium*); and exposed to *P. falciparum* + intestinal protozoa (*C. parvum* and *G. duodenalis*). Significance of differences in seropositivities across sites was assessed using Pearson’s chi-square by default; Fisher’s exact test was used when any expected cell count < 5.

Exposure type	Overall (*N* = 883)	Diourbel (*N* = 347)	Saraya (*N* = 536)	*P*-value
	% (95% CI)	% (95% CI)	% (95% CI)	
*Cryptosporidium parvum* exposure	18.8 (16.2–21.4) (*n* = 166)	19 (14.9–23.1) (*n* = 66)	18.7 (15.4–22.0) (*n* = 100)	0.893
*Giardia duodenalis* exposure	7.4 (5.6–9.1) (*n* = 65)	4.0 (2.0–6.1) (*n* = 14)	9.5 (7.0–12.0) (*n* = 51)	0.002
*Necator americanus* exposure	0.1 (NA)	0 (NA)	0.2 (NA)	1.000^a^
*Schistosoma mansoni* exposure	4.5 (3.2–5.9) (*n* = 40)	2.9 (1.1–4.6) (*n* = 10)	5.6 (3.7–7.5) (*n* = 30)	0.058
*Strongyloides stercoralis* exposure	7.1 (5.4–8.8) (*n* = 63)	8.4 (5.4–11.3) (*n* = 29)	6.3 (4.3–8.4) (*n* = 34)	0.256
*Taenia solium* exposure	5.8 (4.2–7.3) (*n* = 51)	6.6 (4.0–9.2) (*n* = 23)	5.2 (3.3–7.1) (*n* = 28)	0.382
Historical *Plasmodium falciparum* exposure	41.7 (38.4–44.9) (*n* = 368)	29.4 (24.6–34.2) (*n* = 102)	49.6 (45.4–53.9) (*n* = 266)	<0.001
*P. falciparum* partial protection	12.2 (10.1–14.4) (*n* = 108)	12.7 (9.2–16.2) (*n* = 44)	11.9 (9.2–14.7) (*n* = 64)	0.743
Recent *P. falciparum* exposure	11.2 (9.1–13.3) (*n* = 99)	12.7 (9.2–16.2) (*n* = 44)	10.3 (7.7–12.8) (*n* = 55)	0.266
Helminths + *P. falciparum* exposure	9.4 (7.5–11.3) (*n* = 83)	8.9 (5.9–11.9) (*n* = 31)	9.7 (7.2–12.2) (*n* = 52)	0.703
Intestinal protozoa + *P. falciparum* exposure	13.3 (11–15.5) (*n* = 117)	11.2 (7.9–14.6) (*n* = 39)	14.6 (11.6–17.5) (*n* = 78)	0.156
WASH pathogens + *P. falciparum* exposure	18.0 (15.5–20.5) (*n* = 159)	16.1 (12.3–20.0) (*n* = 56)	19.2 (15.9–22.6) (*n* = 103)	0.245

a Fisher’s exact test.

### 3.2 Exposure to multiple pathogens

**Fig. 3:**
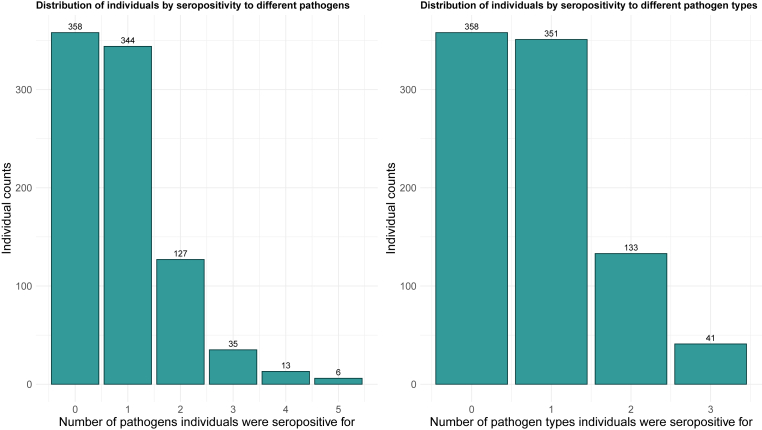
Histogram showing the distribution of individuals based on seropositivity to different pathogens. The y-axis represents the number of individuals. In the left panel, the x-axis indicates the number of distinct pathogens for which individuals tested seropositive (*Plasmodium falciparum*, *Necator americanus*, *Schistosoma mansoni*, *Strongyloides stercoralis*, *Taenia solium*, *Cryptosporidium parvum*, or *Giardia duodenalis*). In the right panel, the x-axis represents the number of pathogen types, grouped into malaria (*Plasmodium falciparum*), helminths (*Necator americanus*, *Schistosoma mansoni*, *Strongyloides stercoralis* and/or *Taenia solium*), and enteric protozoa (*Cryptosporidium parvum*, and/or *Giardia duodenalis*).

Many participants (358/883; 40.5%) were seronegative for all pathogens. Three hundred forty-four (40.0%) were seropositive for one pathogen, 127 (14.4%) for two, 35 (4.0%) for three, 13 (1.5%) for four, and 6 (0.7%) for five. By pathogen type (malaria, helminths, intestinal protozoa), 351/883 (39.8%) were seropositive for one type, 133 (15.1%) for two, and 41 (4.6%) for all three types (Fig. 3).

**Fig. 4:**
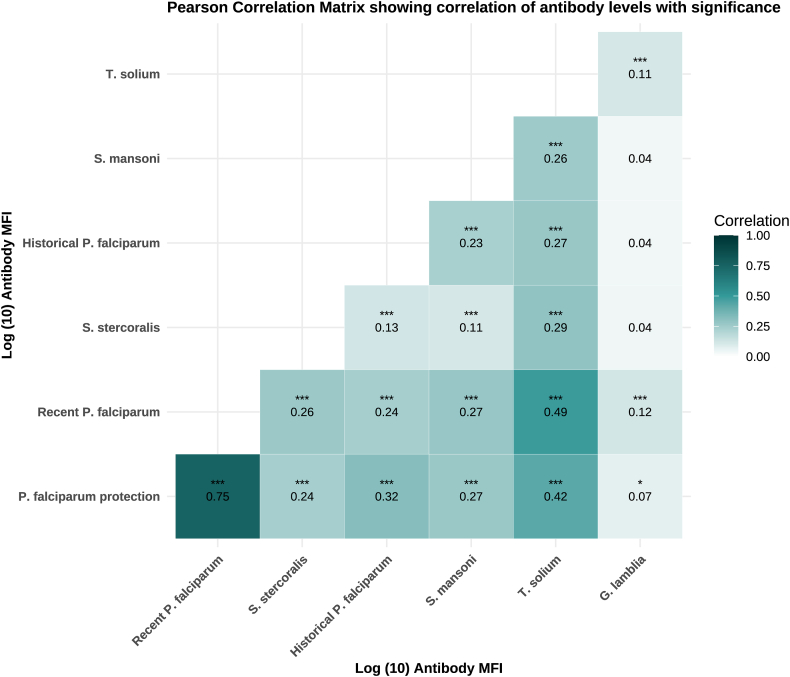
Pearson correlation coefficients quantifying the strength and direction of relationships between antibody levels representative of different exposure types, with values ranging from 0 to 1. The correlation coefficients indicate the degree of association, with values closer to 1 representing strong positive correlations and values closer to 0 indicating weak or no correlations. Statistical significance is denoted by asterisks: no asterisk represents non-significant *P*-values ≥ 0.05, ∗*P* < 0.05, ∗∗*P* < 0.01 and ∗∗∗*P* < 0.001.

Antibody levels to *P. falciparum* antigens associated with protective immunity and recent exposure were positively correlated with antibodies to all helminths and intestinal protozoa (*P* < 0.05). Correlations with *C. parvum* and *G. duodenalis* were significant but weaker than those with helminths (Fig. 4). Antibodies indicative of historical *P. falciparum* exposure correlated with all helminths but not with *C. parvum* or *G. duodenalis* (Fig. 4).

Co-exposures of *P. falciparum* with helminths occurred in 9.4% (95% CI: 7.5–11.3 %) of participants, with any intestinal protozoa - in 13.3% (95% CI: 11.0–15.5%), and with at least one WASH-associated pathogen - in 18% (95% CI: 15.5–20.5%) (Table 1).

School-aged children had higher seropositivity for co-exposure to *P. falciparum* and helminths than pre-school-aged children (Supplementary Table S3). No significant differences were observed by sex (Supplementary Table S4) or study site (Table 1).

### 3.3 Factors influencing the seroprevalences to individual pathogens and multiple pathogens

In multivariable analysis of environmental determinants, school-aged children (*vs* preschool-aged) had higher odds of seropositivity for *P. falciparum* partial-protection (OR: 5.5, 95% CI: 3.1–10.5) and historical exposure (OR: 4.2, 95% CI: 2.9–6.1), as well as higher odds of co-exposure of *P. falciparum* with helminths (OR: 3.2, 95% CI: 1.7–6.0) and with WASH-associated pathogens (OR: 2.4, 95% CI: 1.6–3.8) (Supplementary Table S5).

Compared with Diourbel, residence in Saraya was associated with higher odds of seropositivity for historical *P. falciparum* exposure (OR: 7.4, 95% CI: 4.0–14.0), co-exposure of *P. falciparum* with WASH-associated pathogens (OR: 4.8, 95% CI: 2.2–10.5), and co-exposure of *P. falciparum* with intestinal protozoa (OR: 5.1, 95% CI: 2.2–12.1) (Supplementary Table S5).

Infrequent handwashing (rarely/never performing handwashing before meals) was associated with higher odds of seropositivity to *C. parvum* (OR: 3.8, 95% CI: 2.3–6.3), recent *P. falciparum* exposure (OR: 3.8, 95% CI: 2.3–6.3), historical *P. falciparum* exposure (OR: 2.9, 95% CI: 1.7–5.0), co-exposure of *P. falciparum* with WASH-associated pathogens (OR: 4.5, 95% CI: 2.4–8.4), and co-exposure of *P. falciparum* with intestinal protozoa (OR: 3.8, 95% CI: 2.0–7.5) (Supplementary Table S5). In contrast, longer travel time to a water source (≥ 30 min *vs* < 10 min) and infrequent contact with water bodies (never/rarely/monthly *vs* daily/weekly) were associated with lower odds of recent *P. falciparum* seropositivity (OR: 0.3, 95% CI: 0.2–0.5) and of *P. falciparum*-helminth co-exposure (OR: 0.3, 95% CI: 0.2–0.5), respectively (Supplementary Table S5). Finally, open eaves or gaps between the walls and the roof were associated with lower odds of *P. falciparum*-helminth co-exposure (OR: 0.3, 95% CI: 0.2–0.6) (Supplementary Table S5).

## 4 Discussion

### 4.1 Parasite exposure in Diourbel and Saraya

This study measured seropositivity based on IgG levels as markers of exposure to malaria, helminths, and intestinal protozoa among children in Senegal’s Diourbel and Saraya districts, where the elimination of parasitic diseases is progressing more slowly compared to the national average (SMO, 2022). Compared to earlier qPCR-based findings, the serological data revealed higher prevalence for both single and multiple pathogen exposures (Afolabi et al., 2023b), highlighting the value of serology in capturing a broader historical exposure profile not detected by point-in-time diagnostics (Smith et al., 2024).

Age was a key determinant of exposure risk, with school-aged children showing higher seroprevalence for most pathogens than pre-school-aged children (Fig. 2; Supplementary Table S3). While this pattern is compatible with greater cumulative exposure, elevated seropositivity for recent *P. falciparum* exposure (Etramp5 Ag1, GEXP18 (Smith et al., 2024)) in school children suggests that context-specific behaviours may increase vector contact in older school-aged children. Later bedtimes, heat discomfort, and prioritisation of insecticide-treated nets for under-fives and pregnant women can leave older children unprotected. Pupils at Qur’anic schools may sleep in crowded, open-air dormitories where nets are impractical. Night-time socialising, evening chores, and seasonal mobility (for example, accompanying pastoralist or mining families) may further increase exposure to mosquito bites in older children. Alternative explanations cannot be excluded, including age-dependent antibody kinetics, differences in SMC adherence, and unmeasured confounding (for example, household crowding or mobility). Conversely, *G. duodenalis* seropositivity was higher among pre-school-aged children, as shown in other studies (Khattak et al., 2023), potentially reflecting immature immune responses or less awareness of hygiene practices in infants. These findings highlight the need to tailor interventions to both age groups, balancing school-based programmes with targeted measures for younger children, who are particularly vulnerable to severe outcomes such as diarrheal diseases.

Geographical differences in exposure were observed, with higher seropositivity for *G. duodenalis* and *P. falciparum* in Saraya than in Diourbel (Table 1). This pattern may reflect Saraya’s more limited access to electricity, private toilets, and water sources (Supplementary Table S1), consistent with socio-economic and infrastructural factors that can influence pathogen exposure. For *P. falciparum*, historical seropositivity was higher in Saraya, whereas seropositivity for recent exposure did not differ substantially between sites (Table 1), suggesting a historically greater malaria burden rather than ongoing transmission differences. Direct comparisons are constrained by sampling biases in Diourbel’s geographical and demographic coverage (Fig. 1; Supplementary Table S1). Despite this, Saraya remains the primary setting for integrated intervention implementation, as the trial assessing SMC efficacy and safety with anthelmintics was conducted there (Afolabi et al., 2023a, 2023c). These results therefore provide baseline indicators for evaluating intervention effectiveness in Saraya and may guide context-specific strategies in similar epidemiological settings.

The observed correlations among antibody levels for malaria, helminths, and enteric protozoa suggest that shared behavioral and environmental factors contribute to co-exposure (Fig. 4). In our analysis, regular handwashing before meals was protective against seropositivity to multiple pathogens (Supplementary Table S5). This supports findings from Mozambique, where lower helminth and *P. falciparum* IgG levels – as well as total IgE levels – were associated with improved sanitation and socio-economic conditions **(**Santano et al., 2021). Other studies have similarly linked enteric infections to poor hygiene (Kombo Mpindou et al., 2021).

A study in Ethiopia reported that longer distances to water sources were associated with increased helminth prevalence (Phillips et al., 2022), reinforcing the idea that water access facilitates hygiene and reduces infection risk. However, in our study, longer travel times to water sources and infrequent water contact were associated with lower odds of malaria and helminth seropositivity (Supplementary Table S5). This may reflect the dual nature of water sources; while essential for hygiene, public water bodies may serve as breeding habitats for mosquitoes or reservoirs for water-borne pathogen transmission. Alternatively, households farther from water sources may rely more on stored water and reduced environmental contact, or unmeasured socio-economic factors may influence exposure risk. Residual confounding is also possible because households closer to water may differ in unmeasured factors such as socio-economic status, water source type, vector-control coverage, or local ecology that influence exposure risk despite adjustment.

Structural housing features were also associated with infection risk. Children living in dwellings with gaps between the walls and the roof (eaves) had lower odds of co-exposure with *P. falciparum* and helminths (Supplementary Table S5). In our data, these households reported higher ownership and more frequent use of insecticide-treated nets. This pattern is therefore more consistent with mediation by protective behaviour than with structural protection itself. As an alternative explanation, housing type may confound the association: gaps between walls and roof are common in corrugated iron houses, whereas mud houses differ in construction, and these differences may reflect socio-economic conditions and thermal comfort that influence net use. Residual confounding cannot be excluded, including differences in sleeping location or other unmeasured household factors.

### 4.2 Limitations

Although the study combines well-characterised serological markers, rich epidemiological data, and rigorously adjusted regression models, a number of limitations should be acknowledged.

First, sampling in Diourbel was geographically clustered and imbalanced by age and sex (Fig. 1, Supplementary Table S1), which limits site-level comparisons and underpowers several subgroup analyses. In particular, sex- and age-stratified comparisons within Diourbel are vulnerable to sparse counts, especially for rarer helminths such as *N. americanus*. For this reason, estimates for these comparisons should be interpreted with caution. By contrast, Saraya’s sample distribution was more spatially and demographically representative, so our site-specific interpretation focuses primarily on that district.

Secondly, limited antigen coverage constrained our helminth assessment. We could not evaluate exposure to *Ascaris lumbricoides*, *Trichuris trichiura*, or *Schistosoma haematobium* because relevant antigens were unavailable. Moreover, currently available serological assays for these species can show low sensitivity, high background, and cross-reactivity, which may bias total helminth burden and polyparasitism estimates (Else et al., 2020). Consequently, our helminth and co-exposure seroprevalence likely represents a conservative lower bound.

Thirdly, antibody cross-reactivity is a potential source of misclassification. Although conserved targets such as MSP1-19 raise a theoretical risk of cross-species recognition, prior work reported MSP1-19 to be species-specific across human malaria species (Priest et al., 2018). In our study, we reduced cross-reactivity risk by profiling multiple *P. falciparum-*specific markers. Nevertheless, minimal cross-reactivity cannot be completely excluded. In addition, antigens for other malaria species (*P. vivax*, *P. ovale*, and *P. malariae*) were not included. Although these species are uncommon in Senegal (SMO, 2022), their omission could lead to under-representation of non-*falciparum* exposure.

Finally, because samples were collected over two different months, June in Saraya and November in Diourbel, seasonal variation in antibody responses and exposure risk may have introduced residual effects that we could not fully adjust for.

### 4.3 Future directions

Findings from this study provide a foundation for designing integrated strategies to control multiple parasitic infections in Saraya. Although the integration of SMC with anthelmintic treatment has been shown to be safe and acceptable in this district (Afolabi et al., 2023a, 2023c), the low seroprevalence of helminths and *P. falciparum*-helminth co-exposure, consistent with prior qPCR-based results (Afolabi et al., 2023b), raises questions about the added benefit of routine MDA for helminths in this setting. Broader serological surveys, incorporating a wider antigen panel and more representative sampling across districts, are needed to confirm these patterns and guide decisions on targeted *versus* universal drug-based approaches.

In contrast, moderate *P. falciparum* exposure, as indicated by the reported seroprevalences, supports the continuation and possible expansion of SMC programmes. Furthermore, the notably high seroprevalence of *C. parvum* highlights the potential need to incorporate tinidazole into treatment protocols. This drug also targets *G. duodenalis*, which, although not highly prevalent, remains an important cause of morbidity and mortality among pre-school-aged children. Additionally, *Entamoeba histolytica* exposure was not quantified in this study, but could be another major contributor to the enteric pathogen burden and may similarly benefit from treatment with tinidazole. Given the lack of established guidelines, the findings from this study underscore the need for further research to define effective strategies for preventing intestinal protozoa in Senegal.

Beyond drug-based interventions, improving access to clean water, sanitation facilities, and essential infrastructure is critical to address the root causes of exposure and reduce reliance on reactive healthcare measures. By integrating disease-control strategies, progress can be made towards the sustainable elimination of these parasitic diseases in Senegal.

## 5 Conclusions

The detection of malaria, helminths, and intestinal protozoa among children in Saraya and Diourbel suggests co-existing parasite transmission in these districts. The low seroprevalence of helminths suggests that elimination may be achievable with more targeted and locally tailored strategies. In contrast, *P. falciparum* exposure levels support the continuing implementation of SMC to reduce exposure rates among children. Notably, enteric protozoa, particularly *C. parvum*, represent a significant and under-addressed burden, highlighting gaps in current control programmes. These findings, combined with further investigation, are valuable to guide effective and context-specific strategies for the sustainable allocation of resources and eventual elimination of parasitic diseases in Senegal and similar settings.

## Ethical approval

This study involving human participants was reviewed and approved by the London School of Hygiene & Tropical Medicine (LSHTM) Ethics Committee (No. 22823/14 January 2021) and the Comité National d’Éthique pour la Recherche en Santé (CNERS), Senegal (00000056/MCAS/CNERS/SP/27 April 2021). Written informed consent was obtained from the legal guardians or next of kin of all participants.

## CRediT authorship contribution statement

**Helena Brazal Monzó:** Conceptualization, Formal analysis, Methodology, Validation, Visualization, Writing – original draft, Writing – review & editing. **Santiago Rayment Gómez:** Formal analysis, Validation, Visualization, Writing – review & editing. **Doudou Sow:** Resources, Supervision, Writing – review & editing. **Aminata Colle Lo:** Resources, Data curation, Writing – review & editing. **Marie Pierre Diouf:** Resources, Data curation, Writing – review & editing. **Amadou Seck:** Resources, Data curation, Writing – review & editing. Ibrahima Mbaye: Resources, Data curation, Writing – review & editing. **Elhadji Babacar Fall:** Resources, Data curation, Writing – review & editing. **Catriona Patterson:** Methodology, Writing – review & editing. **Seyi Soremekun:** Formal analysis, Writing – review & editing. **Isaac A. Manga:** Resources, Data curation, Writing – review & editing. **Cheikh Cissé:** Resources, Data curation, Writing – review & editing. **Awa Diouf:** Resources, Data curation, Writing – review & editing. **Ndéye Aida Gaye:** Resources, Data curation, Writing – review & editing. **Kevin K.A. Tetteh:** Resources, Writing – review & editing. **Alex Loukas:** Resources, Writing – review & editing. **Brian Greenwood:** Conceptualization, Resources, Supervision, Writing – review & editing. **Jean Louis A. Ndiaye:** Conceptualization, Resources, Supervision, Writing – review & editing. **Chris Drakeley:** Conceptualization, Resources, Supervision, Writing – review & editing. **Muhammed O. Afolabi:** Conceptualization, Funding acquisition, Resources, Supervision, Writing – review & editing.

## Funding

This study was implemented as part of a career development fellowship awarded to Muhammed Afolabi, which is funded under the UK Research and Innovation Future Leaders Fellowship scheme (MR/S03286X/1). The funder had no role in the study design, data collection and analysis, decision to publish, or preparation of the manuscript.

## Declaration of competing interests

The authors declare that they have no known competing financial interests or personal relationships that could have appeared to influence the work reported in this paper.

## Data Availability

The data supporting the conclusions of this article are included in this published article and its supplementary file. The raw data are available upon request.

## Appendix A Supplementary data

The following iare the supplementary data to this article:
