# Supplementary material for: Serological assessment of pediatric parasite exposure in two Senegalese districts using multiplex serology
Source: Curr Res Parasitol Vector Borne Dis. 2025 Sep 19;8:100320. doi: 10.1016/j.crpvbd.2025.100320 (PMC12508848; doi:10.1016/j.crpvbd.2025.100320)
Supplement: Multimedia component 1 [file mmc1.pdf]

Supplementary Table S1. List of number and percentage of independent variables and their levels selected for multiple logistic regression model (overall, in Diourbel and in Saraya).

**Characteristic**	**Overall** (N = 883)	**Diourbel** (N = 347)	**Saraya** (N = 536)
	***n* (%)**	***n* (%)**	***n* (%)**
Age category			
Pre-school children (≤5 years) (Ref)	315 (36%)	17 (4.9%)	298 (56%)
School children (>5 years)	568 (64%)	330 (95%)	238 (44%)
Assigned hand-washing area			
Yes (Ref)	343 (39%)	133 (39%)	210 (40%)
No	531 (61%)	212 (61%)	319 (60%)
Yes	586 (80%)	331 (96%)	255 (66%)
No (Ref)	143 (20%)	15 (4.3%)	128 (34%)
Electricity			
Yes(Ref)	365 (42%)	286 (83%)	79 (15%)
No	515 (59%)	60 (17%)	455 (85%)
Handwashing before eat			
Always (Ref)	545 (62%)	127 (37%)	418 (78%)
Sometimes	182 (21%)	86 (25%)	96 (18%)
Infrequently (Rarely/never)	154 (17%)	134 (39%)	20 (4%)
House ownership			
Own house/share with family (Ref)	767 (87%)	231 (67%)	536 (100%)
Share with other	116 (13%)	116 (33%)	0 (0%)
Handwashing practice			
Water and soap (Ref)	300 (34%)	146 (42%)	154 (29%)
Water only	579 (66%)	200 (58%)	379 (71%)
Motorbike			
Yes (Ref)	400 (45%)	63 (18%)	337 (63%)
No	480 (55%)	282 (82%)	198 (37%)
Net available			
Yes (Ref)	642 (73%)	295 (86%)	347 (65%)
No	232 (27%)	49 (14%)	183 (35%)
Net use			
Every night (Ref)	519 (84%)	218 (76%)	301 (91%)
Never/Rarely	97 (16%)	69 (24%)	28 (9%)
Radio			
Yes (Ref)	619 (70%)	252 (73%)	367 (69%)
No	262 (30%)	95 (27%)	167 (31%)
Sex			
Female (Ref)	329 (37%)	60 (17%)	269 (50%)
Male	552 (63%)	287 (83%)	265 (50%)
Space between the wall and the roof			
Yes	424 (51%)	241 (71%)	183 (38%)
No (Ref)	401 (49%)	99 (29%)	302 (62%)
Time to waterbody			
<10 minutes (Ref)	442 (50%)	147 (42%)	295 (55%)
10-30 minutes	131 (15%)	5 (1.4%)	126 (24%)
>30 minutes	309 (35%)	195 (56%)	114 (21%)
Toilet type			
Private toilet (Ref)	239 (27%)	162 (47%)	77 (14%)
Shared toilet	591 (67%)	183 (53%)	408 (84%)
Water contact			
Frequently (Weekly/daily) (Ref)	279 (32%)	2 (0.6%)	277 (52%)
Infrequently (Never/rarely/monthly)	604 (68%)	345 (99.4%)	259 (48.3%)
Water source			
Private (Ref)	318 (68%)	262 (98%)	56 (28%)
Public	151 (32%)	4 (1.5%)	147 (72%)

Abbreviations: Ref, reference level used for logistic multiple regression model; *N*, total number of participants.

Supplementary Table S2. Criteria used to calculate antibody levels and seropositivity for each antigen pathogen.

Pathogen type	Antibody levels criterion	Seropositivity criterion
Necator americanus	Mean Na260[1], Na286[1] and Na700[1] MFIs, log scale	Seropositive for at least two of the antigens
Schistosoma mansoni	Sm25[2] MFI, log scale	Seropositive to Sm25
Strongyloides stercoralis	NIE[2] MFI, log scale	Seropositive to NIE
Taenia solium	Mean rES33[2] and T24H[2] MFIs, log scale	Seropositive for either rES33or T24H
Cryptosporidium parvum	Mean CP3[2] and CP17[2] MFIs, log scale	Seropositive for either CP13 or Cp17
Giardia duodenalis	VSP3[2] MFI, log scale	Seropositive for VSP3
P. falciparum historical seropositivity	Mean PfMSP1_19[3], PfAMA1[3] and GLURP R2[3] MFIs, log scale	Seropositivity for at least two of the following antigens: PfMSP1_19, PfAMA1 and GLURP R2
P. falciparum recent seropositivity	Mean Gexp18[3] and Etramp5 Ag 1[3] MFIs, log scale	Either seropositive for Gexp18 or Etramp5 Ag 1
P. falciparum seroprotection	Mean EBA175 RIII-V[3], EBA181 RIII-V[3], Rh2_2030[3], Rh4.2[3] and Rh5.1[3] MFIs, log scale	Seropositive for either EBA175 RIII-V or EBA181 RIII-V and all reticulocyte binding proteins (Rh2_2030, Rh4.2 and Rh5.1)
Internal control for cross reactivity with GST-tagged proteins	GST[3] MFIs	NA
Internal control for total IgG content	Dip-Tox[2] and Tet Tox[4] MFIs	NA

CP17 = Cryptosporidium parvum cp17 gene, CP3 = Cryptosporidium parvum cp23 gene, GST= glutathione-S-transferase, Dip.Tox= Diphtheria Toxoid, EBA175 RIII-V = Erythrocyte binding antigen-175, EBA181 RIII-V = Erythrocyte binding antigen-181, Etramp5 Ag1 = Early transcribed membrane protein 5 antigen (exon) 1, Gexp18 = Plasmodium exported protein, GLURP R2 = Glutamate-rich protein, Na260 = Necator americanus 260 protein, Na286 = Necator americanus 286 protein, Na700 = Necator americanus 700 protein, NIE = Strongyloides stercoralis L3-stage larvae recombinant immunodiagnostic antigen (NIE), MFI= Median Fluorescent Intensity, PfAMA1 = Apical membrane antigen 1, PfMSP1_19 = Merozoite surface protein 1-19, rES33 = taeniasis-specific excretory antigen, Rh2_2030 = Reticulocyte binding protein 2 homologue a, Rh4.2 = Reticulocyte binding protein homologue 4 (RH4), Rh5.1 = Reticulocyte binding protein homologue 5, Sm25 = Schistosoma mansoni Sm25 Protein, Tet.Tox= = Tetanus Toxoid, VSP3 = Giardia duodenalis variant-specific surface protein 3.

[1]Expressed at James Cook University in Australia
[2] Expressed at United States Centers for Disease Control and Prevention
[3] Expressed at the London School of Hygiene & Tropical Medicine
[4]Sourced from NIBSC Reference reagents (Tetanus Toxoid (Non-Adsorbed) 02/232)

Supplementary Table S3. Summary of participants who tested seropositive to single pathogen types and who were exposed to more than one pathogen type by age groups. Groups of co-exposure were: Exposed to *P. falciparum*+WASH-related pathogens (*N. americanus, S. mansoni, S. stercolis, T. solium, C. parvum* and *G. duodenalis*); exposed to *P. falciparum*+ helminths (*N. americanus, S. mansoni, S. stercolis, T. solium)*, and exposed to *P. falciparum* + intestinal protozoa (*C. parvum* and *G. duodenalis*). Significance of differences in seropositivity across sites was assessed using Pearson chi-square test by default; Fisher's exact test was used when any expected cell count was < 5.

**Characteristic**	**Overall** (N = 883)	**Pre-school children (< 5)** (N = 268)	**School children(> 5)** (N = 615)	***P*–value**[1]
	**% (95% CI)**	**% (95% CI)**	**% (95% CI)**	
C. parvum seropositivity	18.8 (16.2–21.4) (*n* = 166)	14.9 (10.7–19.2) (*n* = 40)	20.5 (17.3–23.7) (*n* = 126)	0.052
G. duodenalis seropositivity	7.4 (5.6–9.1) (*n* = 65)	14.6 (10.3–18.8) (*n* = 39)	4.2 (2.6–5.8) (*n* = 26)	<0.001
N. americanus seropositivity	0.1 (NA)	0 (NA)	0.2 (NA)	1.000†
S. mansoni seropositivity	4.5 (3.2–5.9) (*n* = 40)	3.0 (0.9–5.0) (*n* = 8)	5.2 (3.4–7.0) (*n* = 32)	0.148
S. stercoralis seropositivity	7.1 (5.4–8.8) (*n* = 63)	3.4 (1.2–5.5) (*n* = 9)	8.8 (6.5–11.0) (*n* = 54)	0.004
T. solium seropositivity	5.8 (4.2–7.3) (*n* = 51)	4.5 (2.0–7.0) (*n* = 12)	6.3 (4.4–8.3) (*n* = 39)	0.290
Historical *P. falciparum* exposure	41.7 (38.4–44.9) (*n* = 368)	32.5 (26.9–38.1) (*n* = 87)	45.7 (41.8–49.6) (*n* = 281)	<0.001
P. falciparum partial protection	12.2 (10.1–14.4) (*n* = 108)	4.1 (1.7–6.5) (*n* = 11)	15.8 (12.9–18.7) (*n* = 97)	<0.001
Recent *P. falciparum* exposure	11.2 (9.1%–13.3) (*n* = 99)	8.6 (5.2–11.9) (*n* = 23)	12.4 (9.8–15.0) (*n* = 76)	0.102
Helminths+*P. falciparum* seropositivity	9.4 (7.5–11.3) (*n* = 83)	5.6 (2.8–8.3) (*n* = 15)	11.1 (8.6–13.5) (*n* = 68)	0.010
Intestinal protozoa+*P. falciparum* seropositivity	13.3 (11–15.5) (*n* = 117)	10.8 (7.1–14.5) (*n* = 29)	14.3 (11.5–17.1) (*n* = 88)	0.154
WASH pathogens+*P. falciparum* seropositivity	18.0 (15.5–20.5) (*n* = 159)	13.8 (9.7–17.9) (*n* = 37)	19.8 (16.7–23.0) (*n* = 122)	0.033

[1]Pearson chi-square test by default; Fisher's exact test when any expected cell count < 5. Rows where Fisher's exact test was used are marked with †

Supplementary Table S4. Summary of participants who tested seropositive to single pathogen types and who were exposed to more than one pathogen type by sex reported by the legal tutors. Groups of co-exposure were: Exposed to *P. falciparum*+WASH-related pathogens (*N. americanus, S. mansoni, S. stercolis, T. solium, C. parvum* and *G. duodenalis*); exposed to *P. falciparum*+ helminths (*N. americanus, S. mansoni, S. stercolis, T. solium)*, and exposed to *P. falciparum* + intestinal protozoa (*C. parvum* and *G. duodenalis*). Significance of differences in seropositivities across sites was assessed using Pearson chi-square by default; Fisher's exact when any expected cell count < 5.

**Characteristic**	**Overall** (N = 883)	**Female** (N = 329)	**Male** (N = 552)	***P*–value**[1]
C. parvum seropositivity	18.8 (16.2–21.4) (*n* = 166)	19.5 (15.2–23.7) (*n* = 64)	18.5 (15.2–21.7) (*n* = 102)	0.714
G. duodenalis seropositivity	7.4 (5.6–9.1) (*n* = 65)	9.1 (6.0–12.2) (*n* = 30)	6.3 (4.3–8.4) (*n* = 35)	0.123
N. americanus seropositivity	0.1 (NA)	0 (0.0–1.8) (*n* = 0)	0.2 (0.01–1.0) (*n* = 1)	1.000†
S. mansoni seropositivity	4.5 (3.2–5.9) (*n* = 40)	4.3 (2.1–6.4) (*n* = 14)	4.7 (2.9–6.5) (*n* = 26)	0.780
S. stercoralis seropositivity	7.1 (5.4–8.8) (*n* = 63)	4.6 (2.3–6.8) (*n* = 15)	8.7 (6.3–11.0) (*n* = 48)	0.023
T. solium seropositivity	5.8 (4.2–7.3) (*n* = 51)	4.3 (2.1–6.4) (*n* = 14)	6.7 (4.6–8.8) (*n* =3 7)	0.140
Historical *P. falciparum* exposure	41.7 (38.4–44.9) (*n* = 368)	44.1 (38.7– 49.4) (*n* = 145)	40.0 (35.9–44.1) (*n* = 221)	0.240
P. falciparum partial protection	12.2 (10.1–14.4) (*n* = 108)	12.5 8.9–16.0) (*n* = 41)	12.1 (9.4–14.9) (*n* = 67)	0.859
Recent *P. falciparum* exposure	11.2 (9.1%–13.3) (*n* = 99)	11.2 (7.8–14.7) (*n* = 37)	11.2 (8.6–13.9) (*n* = 62)	0.992
Helminths+*P. falciparum* seropositivity	9.4 (7.5–11.3) (*n* = 83)	7.6 (4.7–10.5) (*n*=25)	10.5 (7.9–13.1) (*n* = 58)	0.153
Intestinal protozoa+*P. falciparum* seropositivity	13.3 (11–15.5) (*n* = 117)	14.9 (11.0–18.7) (*n*=49)	12.3 (9.6–15.1) (*n* = 68)	0.271
WASH pathogens+*P. falciparum* seropositivity	18.0 (15.5–20.5) (*n* = 159)	17.9 (13.8–22.1) (*n*=59)	18.1 (14.9–21.3) (*n* = 100)	0.929

[1]Pearson chi-square test by default; Fisher's exact test when any expected cell count < 5. Rows where Fisher's exact test was used are marked with †

Supplementary Table S5. Results from logistic multiple regression, including explanatory variables selected after backward deletion using the Akaike Information Criterion. One logistic regression was run per outcome variable. Significance test is expressed as crude *P*-values ("*P*") and as *P*-values after multiple correction using Holm-Bonferroni method ("$P_{\text{multiple correction}}$"). *P*-values < 0.05 are highlighted in red.

Explanatory variable	Level	Odds ratio	Standard error	95% CI	Outcome variable	*P*	$P_{\text{multiple correction}}$
Age category	School children (>5 years)	1.62	0.21	1.07–2.49	*C. parvum* seropositivity	0.02	1.00
Age category	School children (>5 years)	0.37	0.30	0.20–0.65	*G. duodenalis* seropositivity	0.00	0.10
Age category	School children (>5 years)	3.16	0.32	1.73–6.04	Helminths+*P. falciparum* seropositivity	0.00	0.04
Age category	School children (>5 years)	4.15	0.19	2.88–6.05	Historical *P. falciparum* exposure	0.00	0.00
Age category	School children (>5 years)	5.51	0.31	3.06–10.45	*P. falciparum* partial protection	0.00	0.00
Age category	School children (>5 years)	2.20	0.26	1.34–3.67	Intestinal protozoa+*P. falciparum* seropositivity	0.00	0.25
Age category	School children (>5 years)	2.77	0.40	1.31–6.27	*S. mansoni* seropositivity	0.01	1.00
Age category	School children (>5 years)	3.90	0.39	1.89–8.88	*S. stercoralis* seropositivity	0.00	0.06
Age category	School children (>5 years)	2.41	0.23	1.55–3.81	WASH pathogens+*P. falciparum* seropositivity	0.00	0.02
Sex	Male	1.93	0.33	1.03–3.77	*S. stercoralis* seropositivity	0.05	1.00
Eat soil	Yes	1.76	0.30	1.00–3.22	*C. parvum* seropositivity	0.06	1.00
Eat soil	Yes	4.07	0.54	1.60–13.81	*G. duodenalis* seropositivity	0.01	0.94
Eat soil	Yes	2.93	0.36	1.49–6.26	Intestinal protozoa+*P. falciparum* seropositivity	0.00	0.36
Electricity	No	1.90	0.25	1.17–3.13	*C. parvum* seropositivity	0.01	1.00
Electricity	No	2.25	0.36	1.15–4.67	*G. duodenalis* seropositivity	0.02	1.00
Electricity	No	2.03	0.34	1.04–3.99	*S. stercoralis* seropositivity	0.04	1.00
Handwashing before eat	Rarely/Never	3.76	0.26	2.28–6.25	*C. parvum* seropositivity	0.00	0.00
Handwashing before eat	Rarely/Never	2.18	0.36	1.07–4.35	Helminths+*P. falciparum* seropositivity	0.03	1.00
Handwashing before eat	Rarely/Never	2.93	0.27	1.74–5.00	Historical *P. falciparum* exposure	0.00	0.01

Explanatory variable	Level	Odds ratio	Standard error	95% CI	Outcome variable	*P*	$P_{multiple\ correction}$
Handwashing before eat	Rarely/Never	3.84	0.33	2.00–7.35	Recent *P. falciparum* exposure	0.00	0.01
Handwashing before eat	Rarely/Never	3.81	0.34	1.97–7.48	Intestinal protozoa+*P. falciparum* seropositivity	0.00	0.01
Handwashing before eat	Rarely/Never	2.50	0.34	1.27–4.91	*S. stercoralis* seropositivity	0.01	0.82
Handwashing before eat	Rarely/Never	4.49	0.32	2.43–8.44	WASH pathogens+*P. falciparum* seropositivity	0.00	0.00
Handwashing before eat	Rarely/Never	1.36	0.25	0.83–2.19	*C. parvum* seropositivity	0.22	1.00
Handwashing before eat	Rarely/Never	0.90	0.35	0.44–1.75	Helminths+*P. falciparum* seropositivity	0.76	1.00
Handwashing before eat	Rarely/Never	1.36	0.21	0.90–2.06	Historical *P. falciparum* exposure	0.15	1.00
Handwashing before eat	Rarely/Never	1.16	0.31	0.63–2.09	Recent *P. falciparum* exposure	0.63	1.00
Handwashing before eat	Rarely/Never	1.12	0.29	0.62–1.97	Intestinal protozoa+*P. falciparum* seropositivity	0.70	1.00
Handwashing before eat	Rarely/Never	1.10	0.39	0.49–2.32	*S. stercoralis* seropositivity	0.80	1.00
Handwashing before eat	Rarely/Never	1.21	0.26	0.72–2.01	WASH pathogens+*P. falciparum* seropositivity	0.47	1.00
Handwashing area	No	1.10	0.29	0.63–1.95	*G. duodenalis* seropositivity	0.74	1.00
Handwashing area	No	2.57	0.48	1.07–7.21	*S.mansoni* seropositivity	0.05	1.00
House ownership	Share with others (other than family)	1.98	0.37	0.95–4.13	Helminths+*P. falciparum* seropositivity	0.07	1.00
House ownership	Share with others (other than family)	2.06	0.29	1.16–3.67	Historical *P. falciparum* exposure	0.01	1.00
House ownership	Share with others (other than family)	2.13	0.40	0.97–4.72	*P. falciparum* partial protection	0.06	1.00
House ownership	Share with others (other than family)	3.06	0.38	1.46–6.58	Intestinal protozoa+*P. falciparum* seropositivity	0.00	0.39
House ownership	Share with others (other than family)	2.97	0.47	1.20–7.81	*T.solium* seropositivity	0.02	1.00
House ownership	Share with others (other than family)	3.12	0.34	1.62–6.13	WASH pathogens+*P. falciparum* seropositivity	0.00	0.10
Motorbike	No	1.99	0.25	1.22–3.30	Recent *P. falciparum* exposure	0.01	0.73
Net available	No	0.79	0.21	0.52–1.20	*C. parvum* seropositivity	0.28	1.00

Explanatory variable	Level	Odds ratio	Standard error	95% CI	Outcome variable	*P*	$P_{\text{multiple correction}}$
Net used	Never/rarely	0.66	0.40	0.30–1.44	*P. falciparum* partial protection	0.30	1.00
Net used	Never/rarely	0.37	0.64	0.09–1.21	*S. mansoni* seropositivity	0.12	1.00
Radio	No	1.82	0.20	1.23–2.68	*C. parvum* seropositivity	0.00	0.29
Radio	No	1.13	0.29	0.63–1.99	*G. duodenalis* seropositivity	0.68	1.00
Radio	No	1.71	0.18	1.20–2.43	Historical *P. falciparum* exposure	0.00	0.33
Radio	No	1.79	0.25	1.08–2.93	*P. falciparum* partial protection	0.02	1.00
Radio	No	2.02	0.23	1.30–3.15	Intestinal protozoa+*P. falciparum* seropositivity	0.00	0.21
Radio	No	1.81	0.21	1.19–2.73	WASH pathogens+*P. falciparum* seropositivity	0.00	0.55
Space between the wall and roof	Yes	0.66	0.20	0.44–0.98	*C. parvum* seropositivity	0.04	1.00
Space between the wall and roof	Yes	0.32	0.29	0.18–0.55	Helminths+*P. falciparum* seropositivity	0.00	0.01
Space between the wall and roof	Yes	0.66	0.26	0.39–1.10	*P. falciparum* partial protection	0.11	1.00
Space between the wall and roof	Yes	0.57	0.25	0.35–0.92	Recent *P. falciparum* exposure	0.02	1.00
Space between the wall and roof	Yes	0.55	0.24	0.34–0.87	Intestinal protozoa+*P. falciparum* seropositivity	0.01	1.00
Space between the wall and roof	Yes	0.27	0.50	0.09–0.68	*S. mansoni* seropositivity	0.01	0.94
Space between the wall and roof	Yes	0.49	0.31	0.27–0.89	*S. stercoralis* seropositivity	0.02	1.00
Space between the wall and roof	Yes	0.40	0.37	0.19–0.81	*T. solium* seropositivity	0.01	1.00
Space between the wall and roof	Yes	0.52	0.22	0.34–0.79	WASH pathogens+*P. falciparum* seropositivity	0.00	0.32
Study Site	Saraya	7.40	0.32	4.00–14.03	Historical *P. falciparum* exposure	0.00	0.00
Study Site	Saraya	1.87	0.39	0.88–4.07	*P. falciparum* partial protection	0.11	1.00
Study Site	Saraya	5.11	0.43	2.24–12.12	Intestinal protozoa+*P. falciparum* seropositivity	0.00	0.02
Study Site	Saraya	0.28	0.64	0.08–0.99	*T.solium* seropositivity	0.05	1.00
Study Site	Saraya	4.75	0.39	2.24–10.47	WASH pathogens+*P. falciparum* seropositivity	0.00	0.01
Time to water	>30 minutes	0.30	0.31	0.16–0.54	Recent *P. falciparum* exposure	0.00	0.01
Time to water	10-30 minutes	0.32	0.42	0.13–0.68	Recent *P. falciparum* exposure	0.01	0.63
Toilet type	Shared toilet	0.58	0.29	0.33–1.04	Helminths+*P. falciparum* seropositivity	0.06	1.00
Toilet type	Shared toilet	0.47	0.22	0.30–0.73	Historical *P. falciparum* exposure	0.00	0.09

Explanatory variable	Level	Odds ratio	Standard error	95% CI	Outcome variable	*P*	$P_{\text{multiple correction}}$
Toilet type	Shared toilet	0.45	0.31	0.24–0.81	*P. falciparum* partial protection	0.01	0.87
Toilet type	Shared toilet	0.40	0.44	0.17–0.97	*S. mansoni* seropositivity	0.04	1.00
Toilet type	Shared toilet	0.59	0.24	0.36–0.95	WASH pathogens+*P. falciparum* seropositivity	0.03	1.00
Handwashing practice	Water only	1.42	0.19	0.98–2.06	Historical *P. falciparum* exposure	0.07	1.00
Handwashing practice	Water only	1.71	0.28	1.00–3.02	*P. falciparum* partial protection	0.06	1.00
Handwashing practice	Water only	1.78	0.27	1.06–3.11	Recent *P. falciparum* exposure	0.03	1.00
Water contact	Infrequent (rarely/never/monthly)	0.68	0.23	0.44–1.06	*C. parvum* seropositivity	0.09	1.00
Water contact	Infrequent (rarely/never/monthly)	0.30	0.30	0.16–0.54	Helminths+*P. falciparum* seropositivity	0.00	0.01
Water contact	Infrequent (rarely/never/monthly)	0.61	0.19	0.42–0.89	Historical *P. falciparum* exposure	0.01	1.00
Water contact	Infrequent (rarely/never/monthly)	0.66	0.26	0.39–1.10	Intestinal protozoa+*P. falciparum* seropositivity	0.11	1.00
Water contact	Infrequent (rarely/never/monthly)	0.28	0.40	0.13–0.61	*S. mansoni* seropositivity	0.00	0.19
Water contact	Infrequent (rarely/never/monthly)	0.26	0.46	0.10–0.59	*T. solium* seropositivity	0.00	0.32
Water contact	Infrequent (rarely/never/monthly)	0.65	0.24	0.40–1.03	WASH pathogens+*P. falciparum* seropositivity	0.07	1.00
Water source	Public	1.46	0.30	0.81–2.63	Historical *P. falciparum* exposure	0.20	1.00
Water source	Public	0.82	0.49	0.31–2.14	*P. falciparum* partial protection	0.69	1.00
Water source	Public	0.92	0.46	0.36–2.18	Recent *P. falciparum* exposure	0.85	1.00
Water source	Public	2.75	0.52	1.00–7.80	*T. solium* seropositivity	0.05	1.00
